# The Chemical Profiling, Docking Study, and Antimicrobial and Antibiofilm Activities of the *Endophytic fungi Aspergillus* sp. AP5

**DOI:** 10.3390/molecules27051704

**Published:** 2022-03-05

**Authors:** Mohamed A. Abdelgawad, Ahmed A. Hamed, AbdElAziz A. Nayl, Mona Shaban E. M. Badawy, Mohammed M. Ghoneim, Ahmed M. Sayed, Hossam M. Hassan, Noha M. Gamaleldin

**Affiliations:** 1 Department of Pharmaceutical Chemistry, College of Pharmacy, Jouf University, Sakaka 72341, Aljouf, Saudi Arabia; 2 Microbial Chemistry Department, National Research Centre, 33 El-Buhouth Street, Dokki, Giza 12622, Egypt; ahmedshalbio@gmail.com; 3 Department of chemistry, College of Science, Jouf University, Sakaka 72341, Aljouf, Saudi Arabia; aanayel@ju.edu.sa; 4 Department of Microbiology and Immunology, Faculty of Pharmacy (Girls), Al-Azhar University, Cairo 11754, Egypt; monabadawe1657.el@azhar.edu.eg; 5 Department of Pharmacy Practice, College of Pharmacy, AlMaarefa University, Ad Diriyah 13713, Riyadh, Saudi Arabia; mghoneim@mcst.edu.sa; 6 Department of Pharmacognosy, Faculty of Pharmacy, Nahda University, Beni-Suef 62513, Egypt; ahmed.mohamed.sayed@nub.edu.eg; 7 Department of Pharmacognosy, Faculty of Pharmacy, Beni-Suef University, Beni-Suef 62513, Egypt; 8 Department of Microbiology, Faculty of Pharmacy, The British University in Egypt (BUE), Cairo 11837, Egypt; noha.gamaleldin@bue.edu.eg

**Keywords:** *Endophytic fungi*, chemical profile, antimicrobial, antibiofilm, molecular docking

## Abstract

Growing data suggest that *Aspergillus niger*, an endophytic fungus, is a rich source of natural compounds with a wide range of biological properties. This study aimed to examine the antimicrobial and antibiofilm capabilities of the *Phragmites australis*-derived endophyte against a set of pathogenic bacteria and fungi. The endophytic fungus *Aspergillus* sp. AP5 was isolated from the leaves of *P. australis*. The chemical profile of the fungal crude extract was identified by spectroscopic analysis using LC-HRESIMS. The fungal-derived extract was evaluated for its antimicrobial activity towards a set of pathogenic bacterial and fungal strains including *Staphylococcus aureus*, *Pseudomonas aeruginosa*, *Proteus vulgaris*, *Klebsiella* sp., *Candida albicans*, and *Aspergillus niger*. Moreover, antibiofilm activity toward four resistant biofilm-forming bacteria was also evaluated. Additionally, a neural-networking pharmacophore-based visual screening predicted the most probable bioactive compounds in the obtained extract. The AP5-EtOAc extract was found to have potent antibacterial activities against *S. aureus*, *E. coli*, and *Klebsiella* sp., while it exhibited low antibacterial activity toward *P. Vulgaris* and *P. aeruginosa* and displayed anticandidal activity. The AP5-EtOAc extract had significant antibiofilm activity in *S. aureus,* followed by *P. aeruginosa.* The active metabolites’ antifungal and/or antibacterial activities may be due to targeting the fungal CYP 51 and/or the bacterial Gyr-B.

## 1. Introduction

Common reed or *Phragmites australis* is one of the most common and widespread plants on the planet [1]. Phragmites are perennial grasses that grow along the edges of lakes, marshes, and tropical wetlands [2]. In Egypt, *P. australis* is found in almost all phytogeographical regions [3,4]. It has been recorded in the saline lakes of Wadi El Natrun, 80 km northwest of Cairo, Egypt, as a depression in the Sahara Desert [5]. All the lakes had pH values ranging from 8.5 to 9.5 and salinity from 283 to 540 g/L [6]. One of the first recorded uses of *p. australis* in popular medicine dates back to ancient Egyptian pharaohs (7300–6000 BP). They utilized it to make baskets, mats, nets, pens, and arrows, and for construction [7].

Previous reports have suggested that many of the endophytic organisms that have symptomatically invaded the plant tissues serve an essential function in defending the plant from external stress and preventing other pathogenic organisms from attacking it [8,9,10,11]. These symbiotic relationships promote the endophytic organisms, e.g., fungi, to produce structurally unique metabolites with broad potential applications in the pharmaceutical industry, such as terpenoids, alkaloids, benzoquinones, flavonoids, phenols, benzopyranones, steroids, tetralones, and xanthones [12,13].

The Aspergillus genus is one of the common *Endophytic fungi* with various biologically active compounds that have been identified and isolated, such as brefeldin A, which has anticancer properties [14]; aspernigrin A and naphthoquinoneimine, which have antifungal properties [15]; asporyzin C with antibacterial activity toward *Escherichia coli*; and antioxidants [16,17]. Endophytic *Aspergillus* sp. has recently attracted the interest of researchers as a promising candidate, as it can produce antimicrobial chemicals, which makes it ideal for the development of new antimicrobial compounds. It contains a large number of bioactive compounds with a variety of biological activities when cultivated under regulated laboratory conditions. For example, two alkaloids, fumigaclavine C and pseurotin A, were previously isolated from an endophytic *Aspergillus* species derived from a medicinal plant, *Bauhinia guianensis*. They showed promising antibacterial activity against a number of pathogenic Gram-positive and Gram-negative bacteria with MIC values ranging from 7.81 to 31.25 µg/mL. Moreover, two new alkaloids, 12β-hydroxy-13α-methoxyverruculogen and 3-hydroxyfumiquinazoline A, were isolated from the fermentation broth of Aspergillus fumigatus, an endophytic fungus isolated from the stem bark of Melia azedarach. Both of these alkaloids showed considerable antifungal activity against a number of pathogenic fungi with MIC values ranging from 6.25 to 50 µg/mL [18].

On the other hand, one type of resistance behavior of bacteria is biofilm formation, which represents a current scientific problem, due to the unique capacity of bacteria to modify their immediate environs through phenotypic plasticity that includes changes in their physiology and resistance to antimicrobial drugs [19]. Biofilm-forming microbes have been linked to a variety of infectious disorders, including otitis media, periodontitis, dental caries, and osteomyelitis, as well as chronic conditions such as cystic fibrosis pulmonary infections [20]. Previously, five prenylated benzaldehyde derivative compounds were isolated for the first time from an endophytic fungus, namely, *Aspergillus amstelodami*, cultivated from the *Ammi majus* medicinal plant: Dihydroauroglaucin (1), tetrahydroauroglaucin (2), 2-(3,6-dihydroxyhepta-1,4-dien-1-yl)-3,6-dihydroxy-5-(dimethylallyl)benzaldehyde (3), isotetrahydroauroglaucin (4), and flavoglaucin (5). These metabolites showed antibiofilm activity against both *Staphylococcus aureus* and *Pseudomonas aeruginosa* [19,20].

The hunt for innovative and effective chemical agents from hidden niches is necessitated by different microorganism strains that resist antibiotics and the need to treat serious medical diseases. Endophytes are being studied as a potential for novel medication development [21].

In this study, the main goal was to isolate and identify the *P. australis*-derived endophyte. Then, after the identification of the endophytic fungus chemical profiling using the LC-HRESI-MS technique, we examined the antimicrobial and antibiofilm capabilities against a set of pathogenic bacteria and fungi. Additionally, neural network pharmacophore-based virtual screening was used to correlate the relationship between the chemical constituents and their antimicrobial and antibiofilm activities.

## 2. Materials and Methods

### 2.1. Sample Collection and Isolation of Endophytic fungi

Healthy *P. australis* samples were gathered from Lake El-Bida in Egypt’s Beheira Governorate’s Wadi El-Natrun depression on 10 June 2017, and the morphological characteristics were identified. *P. australis* leaves were rinsed thoroughly in sterile distilled water before being surface sterilized in 70% ethanol for 1 min, in sterile distilled water for 1 min, and in 2% sodium hypochlorite for 1 min; then, they were rinsed three times in sterile distilled water. Sterilized samples were dried in a laminar flow hood on sterile paper towels, then sliced into tiny segments, and incubated on potato dextrose-sea water agar (PDA-SW), with filter-sterilized nalidixic acid (50 mg/L) and chloramphenicol (200 mg/L) supplements. The plates were incubated at room temperature until the growth of *Endophytic fungi* was observed. Colonies with different morphological traits were chosen and sub-cultured on newly produced PDA-SW without antibiotics to obtain a pure culture, which was then kept at 4 °C. The strain AP5 is housed in Egypt’s National Research Centre (NRC) Microbial Chemistry Department. 

### 2.2. Identification of Fungal Strain

#### 2.2.1. Phenotypic Study 

The fungal isolate was identified utilizing cultural and morphological characteristics such as colony development pattern and conidial morphology, as a starting point [22,23].

#### 2.2.2. Genotypic Study

For further identification confirmation, 18S rDNA sequence analysis of the fungal isolate was performed. For fungal DNA extraction, in a 250 mL Erlenmeyer’s flask with a 50 mL Potato dextrose broth medium, mycelia were inoculated and incubated for 4 days at 28 °C. After the incubation period, the genomic DNA was extracted from the mycelial biomass using the Mini Kit, Qiagen DNeasy LLC, Germantown, USA following the manufacturer’s protocol.

The amplification reactions of the 18S rRNA gene were carried out using 2 universal primers NS3 (5′-GCAAGTCTGGTGCCAGCAGCC amplification. 3′)/NS4 (5′-CTTCCGTCAATTCCTTTAAG-3′) (Lu et al., 1995). The following PCR temperature profile was used: a 5 min denaturation stage at 94 °C, followed by 35 cycles of 94 °C for 30 s, 55 °C for 30 s, 72 °C for 90 s, and a final extension step of 72 °C for 5 min. Electrophoresis was used to assess the amplified products sequenced in SolGent Company, South Korea. The sequence produced was analyzed using the BLASTN program to investigate the 18S rRNA gene sequences’ similarity and homology with similar existing sequences from the NCBI database (National Centre Biotechnology Information, http://www.ncbi.nlm.nih.gov, accessed 21 September 2021). MEGAx software was used to create the phylogenetic tree. The Tamura–Nei model and the Maximum Likelihood approach were used to infer the evolutionary history [24]. The tree with the highest log likelihood (−22,507.44) is shown. Next to the branches is the proportion of trees in which the related taxa are grouped. The initial tree(s) for the heuristic search was automatically generated by applying the Neighbor-Join and BioNJ algorithms to a matrix of pairwise distances computed using the Tamura–Nei model and then selecting the topology with the highest log-likelihood value. The branch lengths were measured by the number of substitutions per site, and the tree is depicted to scale. There were 18 nucleotide sequences in this study; 1st + 2nd + 3rd + noncoding codon locations were included. In all, there were 1504 locations in the final dataset. MEGA X was used to undertake the evolutionary analysis [25].

### 2.3. Cultivation of Endophytic fungi

The seed culture of *Aspergillus* sp. AP5 was established by inoculating the fungal mycelia into a 250 mL Erlenmeyer flask containing 100 mL potato dextrose broth medium and incubating the mixture for 4 days at 28 °C. Then, 5 mL of the broth culture was transferred in a 1 L conical flask containing a rice medium: 100 g commercial rice and 100 mL 50% seawater. The flasks were incubated at 28 °C for 15 days.

### 2.4. Extraction and Isolation

Following incubation, the fermented rice was soaked overnight in ethyl acetate (1:1 *v*/*v*). The EtOAc extract was collected and evaporated at 45 °C in a water bath by a rotating vacuum evaporator at 100 rev/min to remove the solvent; the obtained crude extract was 10.50 g and provided a crude antimicrobial activity test.

### 2.5. Metabolomic Analysis

Following Alhadrami et al., (2021) [26], the obtained extracts were submitted to metabolic analysis using LC-HRESIMS. The LC-HRESIMS technique is detailed on page S2 of the “Supplementary Material File”.

### 2.6. Antimicrobial Activity 

To determine the antimicrobial activity of the cure extract, four Gram-negative bacteria (*Escherichia coli* ATCC 25955, *Pseudomonas aeruginosa* ATCC 10145, *Proteus vulgaris*, and *Klebsiella pneumoniae*), one Gram-positive bacteria (*Staphylococcus aureus* NRRL B-767), and one yeast (*Candida albicans* ATCC 10231) were used as test microbes, and the experiment was performed in 96-well flat polystyrene plates. The plates were incubated overnight at 37 °C with 10 µL of test extracts (final concentration of 500 µg/mL) added to 80 µL of lysogeny broth (LB broth), followed by 10 µL of bacterial culture suspension (log phase). The antibacterial activity of the tested drug was detected as clearing in the wells after incubation, whereas compounds that had no impact on the bacteria caused the growth media to appear opaque in the wells. In a Spectrostar Nano Microplate Reader BMG LABTECH GmbH, Allmendgrun, Germany), the absorbance was measured after roughly 20 h at OD600. 

### 2.7. Antibiofilm Assay

A microtiter plate assay was used to test the antibiofilm forming activity. In a 96-well polystyrene titer plate, the effect of fungal crude extract from fungi on biofilm development was studied [27]. The bacteria were initially inoculated in LB broth in a 100 mL Erlenmeyer flask and incubated at 37 °C overnight at 150 rpm. Briefly, 180 LB broth was poured into each well, subsequently infected with 10 μL of the overnight pathogenic bacterial suspension. Then, 10 μL of the test crude at (500 μg/mL) concentration was added, along with a control (without the test sample), and they were incubated statically at 37 °C for 24 h. The contents of the wells were carefully removed after incubation. The microplate wells were washed three times with 200 µL of distilled water per well to remove free-floating bacteria, air-dried for 1 h, stained with 200 µL per well crystal violet solution (0.1%, *w*/*v*), and left at room temperature for 10 min to remove the excess stain; the microplate wells were then washed three times with 200 µL per well phosphate buffer saline (PBS) pH 7.2 to remove free-floating bacteria and air-dried for 1 h. The dried microplate was rinsed with 200 µL of 95% ethanol in each well to solubilize the dye, and the intensity was measured using a Spectrostar Nano Microplate Reader at optical density 570 nm (BMG LABTECH GmbH, Allmendgrun, Germany).

### 2.8. In Silico Biological Activity Predictions

PASS software was employed to predict the most probable antibacterial and antifungal metabolites in the *Aspergillus* sp. AP5-derived extract. The details for PASS are described in the Supplementary Material File, page S4.

### 2.9. Determination of the Potential Protein Targets of the Annotated Compounds

To determine the potential targets for the dereplicated compounds, we performed inverse docking against all proteins present in PDB. The details are described in the Supplementary Material File, page S4.

## 3. Results and Discussion 

### 3.1. Isolation of the Producing Endophytic fungal Strain

*P. australis* leaves were gathered from Lake El-Bida, Wadi El-Natrun depression, Beheira Governorate, Egypt, to obtain the fungus AP5. The isolated strain was grown on a potato dextrose agar slant containing 50% marine seawater and maintained at −80 °C in 50% glycerol [28]. The strain was deposited in the National Research Centre’s Microbial Chemistry Department in Cairo, Egypt.

### 3.2. Fungal Phenotypic Characteristics 

**Figure 1:**
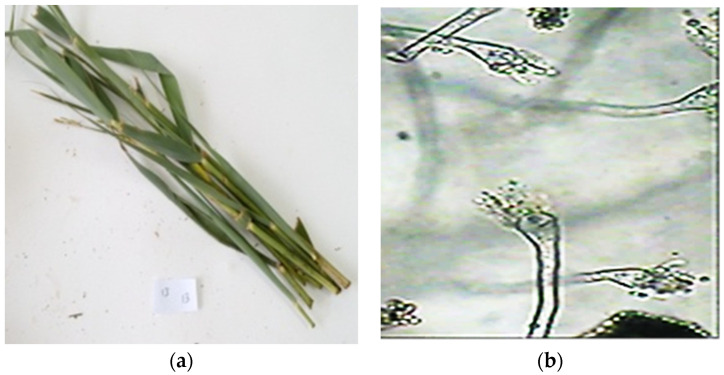
Representative photos of (**a**) *P. australis* leaves and (**b**) mycelium, conidiophores, and conidia as seen under the light microscope.

The fungus AP5 grew to a diameter of 3.0–3.5 cm on Czapek agar at 25 °C in 7 days, giving it a buff to golden brown color. The conidial heads were columnar, and the vesicle was sub-globose and 9.0 μm in diameter. The primary sterigmata diameter was 5.5 × 2.5 μm, while the secondary sterigmata were 4.0 × 2.0 μm in diameter. The diameter of the conidiophore was 3.0 m. The conidia were globose, smooth, and had a diameter of 2.0 m. The morphology results of the isolated fungus confirmed the isolated fungi belonged to *Aspergillus* sp. (Figure 1b).

### 3.3. Molecular Identification

**Figure 2:**
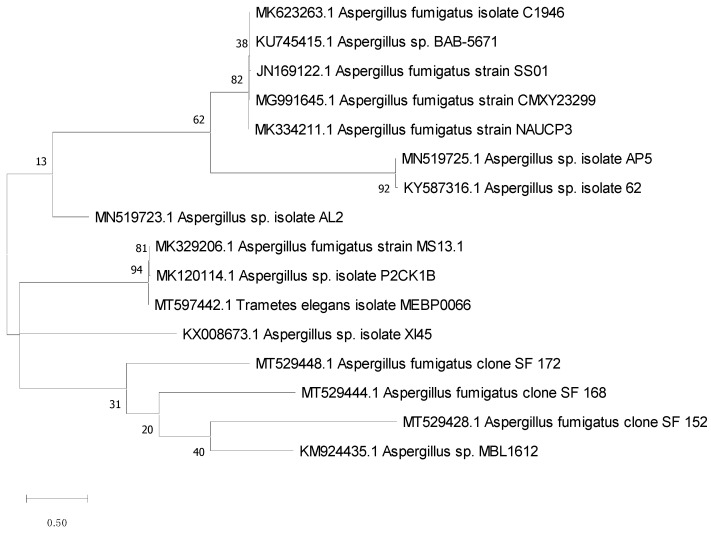
Constructed tree using the Neighbor-Joining method to match the fungus AP5 to already published sequences.

The 18S rRNA gene sequence is a standard molecular marker for biodiversity studies, since it is highly conserved intra-species (similarities close to 100%) and assists in species-level analyses. So, it was retrieved, identified, and matched against other recognized sequences in the GeneBank database using the BLAST program to determine the similarity score and statistical significance of the hits (http://www.blast.ncbi.nlm.nih.gov/Blast, accessed 10 October 2021). With 99.83% homology of the isolate AP5 with *Aspergillus* sp. (Figure 2), the acquired result revealed a close resemblance to the 18S rRNA gene sequence. MEGA x software was used to create the phylogenetic study and tree using the Maximum Likelihood technique according to Kumar et al. (2016) (Figure 2) [29]. The strain AP5 was identified as *Aspergillus* sp. AP5 based on DNA sequence analysis and physical traits, and its accession number MN519725 has been deposited in GenBank. 

### 3.4. Biological Activity

#### 3.4.1. Antimicrobial Potential

The antimicrobial potential of the endophytic fungus *Aspergillus* sp. AP5 crude extract was investigated against a group of microbes using the MTP assay. The results showed that the AP5-EtOAc extract displayed pronounced antimicrobial properties against *S. aureus*, *E. coli*, and *Klebsiella* with inhibition ratios of 53.04%, 61.23%, 51.16%, respectively; although, it had a moderate antimicrobial property against *E. coli* and *Klebsiella* sp. The AP5-EtOAc extract exhibited low antibacterial activity toward *P. Vulgaris* and *P. aeruginosa* with an inhibition ratio of 15.44% and 30.25%, respectively. Additionally. the antifungal activity of AP5-EtOAc was also evaluated against *C. albicans,* and the results showed that both Te-EtOAc and Te-acetone displayed anticandidal activity (Table 1).

#### 3.4.2. Antibiofilm Activity

**Figure 3:**
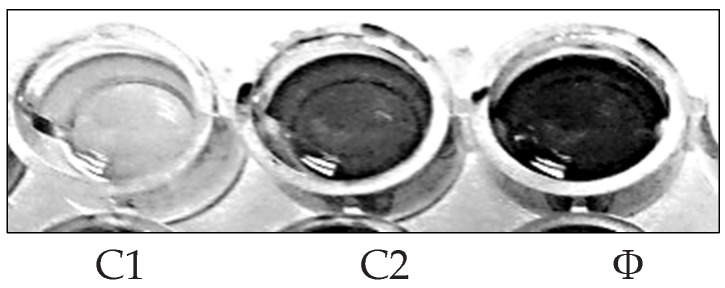
Microtiter plate biofilm alone (Ø), and biofilm inhibition of *S aureus* (C1) and *B. subtilis* (C2).

**Figure 4:**
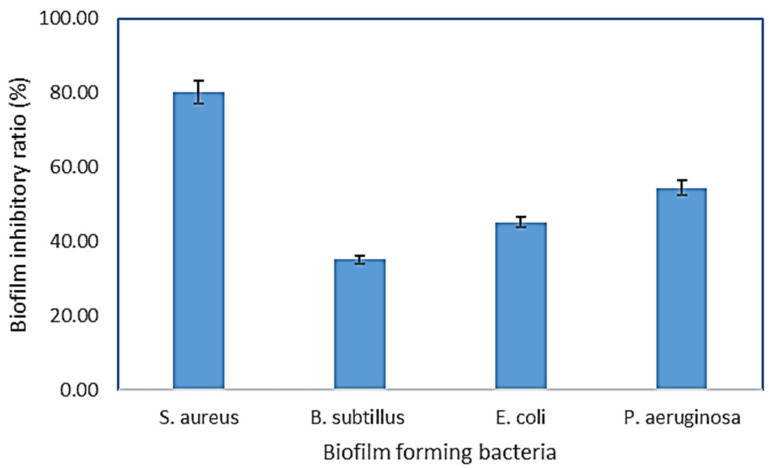
AP5-EtOAc extract inhibits biofilm formation in *P. aeruginosa, S. aureus, E. coli,* and *B. subtilis.* The microtiter plate test and the crystal violet assay measured the biofilm. The graph’s bars show the mean ± SD as a percentage of biofilm inhibition.

A bacterial biofilm is a collection of microorganisms living in extracellular polymeric substances (EPS) that forms during the biofilm’s adhesion to the surface. This natural phenomenon is considered an essential source of nosocomial infections [30]. Using microtiter plates, (Figure 3), biofilm inhibition activity was investigated, the crude extract and purified components were tested for biofilm inhibition efficacy against four clinical pathogenic bacteria (*P. aeruginosa*, *S. aureus*, *E. coli*, and *B. subtilis*), and each of these bacteria’s biofilms was compared to untreated biofilms (control) (Figure 4) The crude extract showed significant antibiofilm activity in preliminary antibiofilm studies against *S. aureus* with biofilm inhibitory up to 80.035%. Additionally, the crude extract reduced the biofilm formation of the strain *P. aeruginosa* by 54.41%, while *B. subtilis* biofilm formation was inhibited up to 35.15% by AP5-EtOAc. The proportion of biofilm inhibition in *E. coli* was the lowest compared to the other test microbes.

### 3.5. LC-HRMS Chemical Profiling

**Figure 5:**
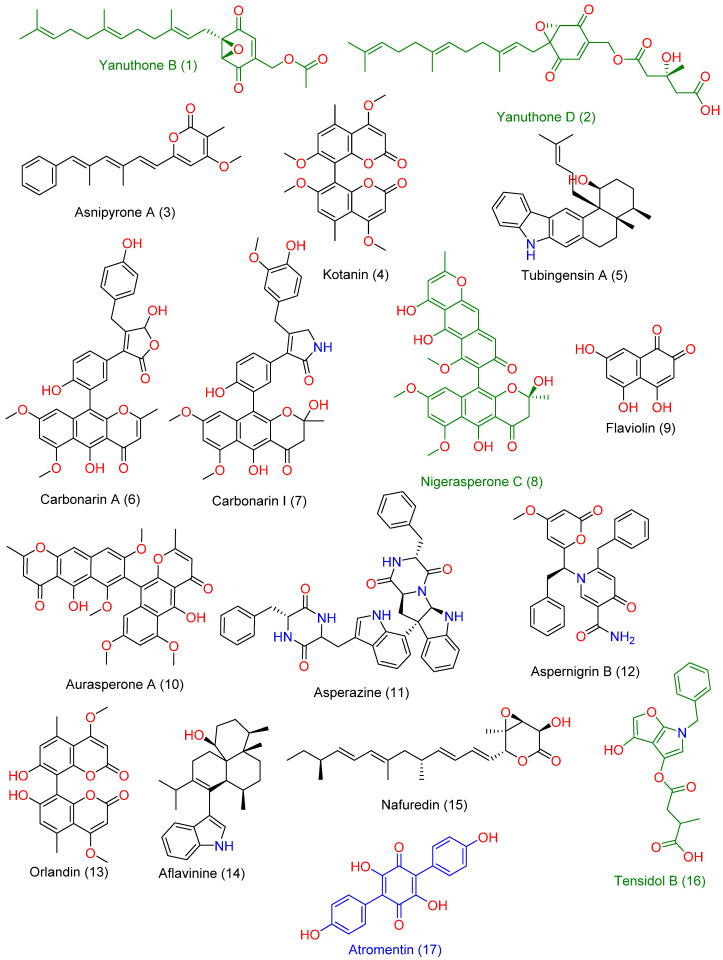
Chemical structures of the compounds **1**–**17** were putatively dereplicated in *Aspergillus* sp. AP5-derived extract. Green-colored compounds were previously reported to have antifungal activity, while blue-colored compound (compound **17**) has been reported to have antibacterial activity.

LC-HRMS-assisted chemical characterization of *Aspergillus* sp. AP5-derived extract led to the annotation of 17 major compounds (Supplementary Material File, Table S1, and Figure 5). The putatively specified compounds were discovered to be members of the polyketide natural product category, including nitrogen-containing ones (compounds **7**, **11**, **12**, and **16**) and indole-containing alkaloids (compounds **5** and **14**). All the chemicals discovered have previously been recognized as Aspergillus metabolites [31,32,33]. 

The following are the previously reported activities of most of them, including antibacterial and antifungal activity: Yanuthone B and D (compounds **1** and **2**) have been reported to exert broad-spectrum antifungal properties [34]. Asnipyrone A (3) has been shown to have a weak cytotoxic effect on several cancer cells lines from humans [35] (Liu et al., 2011). Tubingensin A (5) is an indole-based pentacyclic alkaloid that has been shown to have potent antiviral properties against the Herpes simplex virus [36]. Carbonarin A and I (6) demonstrated moderate in vitro cytotoxicity against K562 cells [37]. Nigerasperone C (8) was shown to have mild antifungal efficacy in vitro [38]. Asperazine (11) is a potent cytotoxic diketopiperazine alkaloid derived from the Aspergillus species with a marine origin [39]. Aspernigrin B (12) has also been extracted from a marine-derived Aspergillus species and has been reported to exert potent in vitro anti-HIV activity [40]. Aflavinine (14) is an indole-based alkaloid with reported antifeedant activity against fungivorous insects [41]. Nafuredin (15) is a unique polyene natural product with a selective NADH-fumarate reductase inhibition activity [42]. Tensidol B (16) is an unusual alkaloid with broad-spectrum antifungal activity [43]. Atromentin (17) has shown potent antibacterial activity against Streptococcus pneumoniae via the inhibition of its enoyl-ACP reductase (FabK) enzyme [44].

### 3.6. Target Prediction and Docking Analysis

Computer-aided biological activity predictions that depend on artificial intelligence have become widely used as a preliminary step in the process of drug discovery. Such virtual and computer-aided procedures could be helpful in the analysis of a natural-crude extract [45,46,47].

Consequently, we subjected the annotated compounds (**1**–**17**) in the *Aspergillus* sp. AP5-derived extract to the neural networking and pharmacophore-based prediction software PASS. This platform has proven successful in its predictions, particularly for those with a probability higher than Pa = 0.5 [26,48,49,50,51,52,53].

**Figure 6:**
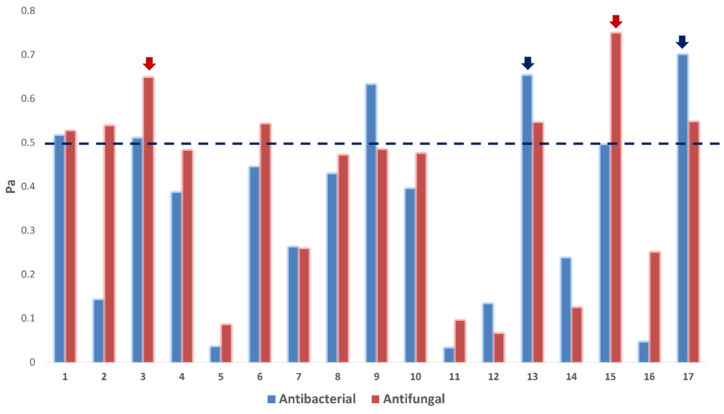
PASS prediction scores of the compounds **1**–**17** as antibacterial and antifungal agents. Pa > 0.5 indicates a high probability of being active in vitro. Red-colored arrows indicate compounds predicted to target C. albicans’ CYP 51. Blue-colored arrows indicate compounds predicted to target *E.* *coli*’s Gyr-B.

As shown in Figure 6, only compounds **1**, **3**, **13**, and **17** obtained Pa scores > 0.5 for antibacterial and antifungal activities. Compounds **2**, **6**, and **15** obtained Pa scores > 0.5 for the antifungal activity only, while compound **9** showed Pa scores > 0.5 for the antibacterial activity only. The remaining compounds were probably inactive as either antibacterial or antifungal agents (i.e., Pa scores < 0.5; Figure 6).

**Figure 7:**
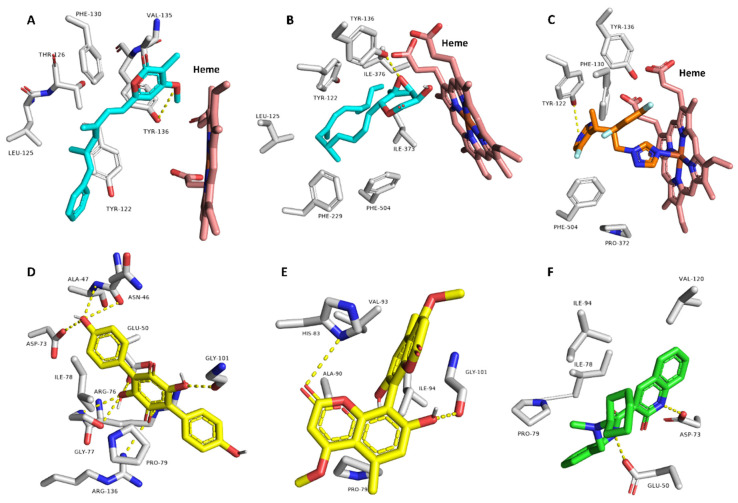
Predicted binding modes of compounds **3** and **15** ((**A**,**B**), respectively) inside the active site of *C. albicans*’s CYP 51 and compounds **13** and **17** inside the active site of *E. coli*’s Gyr-B ((**D**,**E**), respectively), alongside the co-crystallized inhibitors ((**C**,**F**), respectively).

Thereafter, we subjected the potentially active compounds (i.e., with Pa scores > 0.5) to an inverse docking-based virtual screening using idTarget platform [54] to determine the most probable molecular target(s) to mediate their predicted antibacterial and/or antifungal activities. Interestingly, compounds **3** and **15** showed significant binding energy scores (−9.4 and −8.9 kcal/mol) with the 14-α-demethylase (CYP 51) of Candida albicans (PDB: 5TZ1) [47]. Additionally, their binding modes inside the protein’s active site were comparable with that of its co-crystallized inhibitor (Figure 7). They established H-bonds with TYR-136 and many hydrophobic interactions with LEU-125, TYR-122, PHE-130, VAL-135, PHE-229, PHE-504, and ILE-376.

CYP 51 is a key enzyme in the ergosterol (i.e., an essential fungal cell membrane component) biosynthetic pathway and has been studied as a very good target for azole-based compounds [55].

On the other hand, compounds **13** and **17** showed significant binding energy scores (−10.1 and −8.1 kcal/mol) with the DNA gyrase (subunit-B; Gyr-B) of Escherichia coli (PDB: 6KZV) [48].

Gyr-B is the ATP-hydrolyzing unit of the bacterial DNA gyrase responsible for uncoiling DNA [56].

This previous in silico-based screening was carried out to putatively determine the relevant antibacterial and antifungal compounds in the investigated *Aspergillus* sp. AP5-derived extract.

## 4. Conclusions

The growing spread of infectious diseases caused by microbes such as bacteria, viruses, and fungi has become one of the most significant challenges for the future of humanity. The endophytic fungus *Aspergillus* sp. AP5 was studied, because it exhibited intriguing inhibitory effects and antibiofilm activity toward both Gram-positive and Gram-negative bacteria and antifungal activity against some pathogenic fungi. The chemical annotation of this extract using LC-HRESIMS revealed that it was high in bioactive polyketide-based natural compounds. In silico screening of dereplicated metabolites in the fungus extract revealed several compounds that could be potentially active chemical entities. It was further proposed that these active metabolites may have antifungal and/or antibacterial properties by targeting the fungal CYP 51 and/or the bacterial Gyr-B, respectively. 

Further work will have to aim at the purification of these putatively active components from the fungal crude extract, in order to verify the predicted targets and to allow in vitro and in vivo tests with pure single constituents to determine their usefulness as antibacterial agents.

## Figures and Tables

**Table 1:** In vitro antimicrobial (antibacterial and antifungal) activity of *Aspergillus* sp. *AP5* Crude extract.

	Inhibition Ratio (%)						
	Antibacterial					Antifungal	
	*S. aureus*	*P. vulgaris*	*E. coli*	*P. aeruginosa*	*Klebsiella* sp.	*A. niger*	*C. albicans*
**AP5-EtOAc**	53.04 ± 1.51	15.44 ± 4.10	61.23 ± 3.45	30.25 ± 4.88	51.16 ± 2.51	40.25 ± 2.50	40.14 ± 1.22
**Cip**	99.10	95.05	98.22	99.00	95.05	nd	nd
**Nys**	nd	Nd	nd	nd	nd	98.06	99.15

**Cip:** positive antibacterial control Ciprofloxacin 10 µg/mL, control, **Nys**: positive antifungal control Nystatin 10 µg/mL.

## Data Availability

The data presented in this study are available on request from the corresponding author.

## Supplementary Materials

The following are available online, [54]. Table S1. Dereplicated-metabolites-from-LC-HRESIMS analysis of *Aspergillus* sp. AP5 extract.

## Sample Availability

Samples of the compounds are available from the authors, for further questions concerning it kindly contact Hossam M. Hassan.
