# Supplementary material for: The Chemical Profiling, Docking Study, and Antimicrobial and Antibiofilm Activities of the Endophytic fungi Aspergillus sp. AP5
Source: Molecules. 2022 Mar 5;27(5):1704. doi: 10.3390/molecules27051704 (PMC8911721; doi:10.3390/molecules27051704)
Supplement: Supplementary file 1 [file molecules-27-01704-s001.zip › molecules-1545003-supplementary.pdf]

Bioactive natural products from Aspergillus* sp. AP5 isolated from *P. australis

Table S1. Dereplicated metabolites from LC-HRESIMS analysis of *Aspergillus sp.* AP5 extract

**No.**	**Compound Name**	**MF**	**RT(min.)**	**Accurate Mass**
1	Yanuthone B	$C_{24}H3_2O_5$	7.15	400.2250
2	Yanuthone D	$C_{28}H_{38}O_8$	7.23	502.2567
3	Asnipyrone A	$C_{21}H_{22}O_3$	8.09	322.1569
4	Kotanin	$C_{24}H_{22}O_8$	8.29	438.1315
5	Tubingensin A	$C_{28}H_{35}NO$	8.31	401.2719
6	Carbonarin A	$C_{33}H_{26}O_{10}$	8.39	582.1526
7	Carbonarin I	$C_{34}H_{31}NO_{10}$	8.66	613.1948
8	Nigerasperone C	$C_{31}H_{26}O_{11}$	9.21	574.1475
9	Flaviolin	$C_{10}H_6O_5$	9.35	206.0215
10	Aurasperone A	$C_{32}H_{26}O_{10}$	9.46	570.1526
11	Asperazine	$C_{40}H_{36}N_6O_4$	9.57	664.2798
12	Aspernigrin B	$C_{27}H_{24}N_2O_5$	9.59	456.1685
13	Orlandin	$C_{22}H_{18}O_8$	9.83	410.1002
14	Aflavinine	$C_{28}H_{39}NO$	10.28	420.3266
15	Nafuredin	$C_{22}H_{32}O_4$	10.36	360.2301
16	Tensidol B	$C_{18}H_{17}NO_6$	10.34	343.1056
17	Atromentin	$C_{18}H_{12}O_6$	10.32	324.0634

Metabolomic Analysis Procedure

The prepared crude extract was dissolved in methanol to reach a concentration of 1mg/mL for mass spectrometry analysis. An Acquity Ultra Performance Liquid Chromatography system attached to a Synapt G2 HDMS quadrupole time of flight hybrid mass spectrometer (Waters, Milford, the USA) was utilized. Positive and negative ESI ionization modes were employed to get out the high resolution mass spectrometry connected with a spray voltage at 4.5 kV, the capillary temperature at 320℃, and mass range from *m/z* 150–1500. The MS dataset was processed and data were obtained utilizing MZmine 2.20 based on the accepted parameters. Mass ion peaks were identified and accompanied by chromatogram builder and chromatogram deconvolution. The local minimum search algorithm was addressed and isotopes were too analyzed via the isotopic peaks of grouper. Missing peaks were displayed using the gap-filling peak finder. An adduct search along with a complex search was carried out. The processed data set was later exposed to molecular formula prediction and peak identification. The positive and negative ionization mode data sets from the respective extract were dereplicated against the DNP (Dictionary of Natural Products) databases.

In Silico Biological Activity Predictions

PASS was employed for the prediction of the most possible antibacterial and antifungal metabolites in *Aspergillus sp.* AP5 extract. This software was capable of to predict >4000 types of pharmacological and toxicological activities including their mechanism of action, with approximately 85% as acceptable precision, depending on the submitted compound structures that were subsequently screened applying the structure activity relationship database (SARBase). The prediction results were given as probability scores (probably active “Pa” or probably inactive “Pi”). These calculated probability scores were determined by linking the structure and

functional groups features in the tested molecules that matched or mismatched the specific activities recorded in the software associated database. The higher the Pa values, the better acceptable it was for the compound to present the suggested pharmacological activity on a scale of 0–1. Pa values higher than 0.5 mean a high experimental chance of the suggested pharmacological activity.

Determination of the Potential Protein Targets of the annotated Compounds

Potential Protein targets for the compounds with high probability to have either antibacterial or antfungal activity (Pa > 0.5) were proposed by subjecting these compounds to inverse docking against all proteins hosted in Protein Data Bank (PDB; https://www.rcsb.org/). idTarget platform (http://idtarget.rcas.sinica.edu.tw/) was used for this task. This structural-based screening software applies a unique docking approach called divide-and-conquer docking that adaptively builds small overlapping grids to make the searching space on the protein surfaces more constrained, and hence, it can run a huge number of accurate docking experiments in much reduced time [1]. The retrieved results were obtained as a list of binding affinity scores arranged from the heist negative value to the lowest one. We set a binding affinity score of -7 kcal/mol as a cut-off value to select the best targets for the query structures. Considering fungal and bacterial proteins, 2 protein targets (CYP 51 and Gyr B, respectively) were selected. The binding modes of top-scoring compounds were visualized using Pymol software.

References:

1. Wang, J. C., Chu, P. Y., Chen, C. M., & Lin, J. H. (2012). idTarget: a web server for identifying protein targets of small chemical molecules with robust scoring functions and a divide-and-conquer docking approach. *Nucleic acids research*, *40*(W1), W393-W399.
